# Connected Vehicles: Technology Review, State of the Art, Challenges and Opportunities

**DOI:** 10.3390/s21227712

**Published:** 2021-11-19

**Authors:** Ghadeer Abdelkader, Khalid Elgazzar, Alaa Khamis

**Affiliations:** Department of Electrical, Ontario Tech University, Oshawa, ON L1G 0C5, Canada; ghadeer.abdelkader@ontariotechu.ca (G.A.); alaa.khamis@ontariotechu.ca (A.K.)

**Keywords:** connected and autonomous vehicles, vehicular communication, Wi-Fi 6, 5G, connected vehicles applications, V2X, challenges, opportunities

## Abstract

In an effort to reach accident-free milestones or drastically reduce/eliminate road fatalities rates and traffic congestion and to create disruptive, transformational mobility systems and services, different parties (e.g., automakers, universities, governments, and road traffic regulators) have collaborated to research, develop, and test connected vehicle (CV) technologies. CVs create new data-rich environments and are considered key enablers for many applications and services that will make our roads safer, less congested, and more eco-friendly. A deeper understanding of the CV technologies will pave the way to avoid setbacks and will help in developing more innovative applications and breakthroughs. In the CV paradigm, vehicles become smarter by communicating with nearby vehicles, connected infrastructure, and the surroundings. This connectivity will be substantial to support different features and systems, such as adaptive routing, real-time navigation, and slow and near real-time infrastructure. Further examples include environmental sensing, advanced driver-assistance systems, automated driving systems, mobility on demand, and mobility as a service. This article provides a comprehensive review on CV technologies including fundamental challenges, state-of-the-art enabling technologies, innovative applications, and potential opportunities that can benefit automakers, customers, and businesses. The current standardization efforts of the forefront enabling technologies, such as Wi-Fi 6 and 5G-cellular technologies are also reviewed. Different challenges in terms of cooperative computation, privacy/security, and over-the-air updates are discussed. Safety and non-safety applications are described and possible future opportunities that CV technology brings to our life are also highlighted.

## 1. Introduction

According to the World Health Organization (WHO), annual traffic accidents account for 1.3 million, and it is considered the first cause of death among young people aged 5–29 years old [1]. According to WHO statistics, every 24 s, a fatal accident occurs. Even with the presence of many preventive safety measures such as airbags, antilock brakes, and other built-in technologies that can help many people involved in accidents to survive, the number of traffic accidents continues to rise. The impact of traffic accidents is not only restricted to increasing the number of road fatalities and injuries but is considered a prime factor in holding back economic growth. The economic losses and monetary burden associated with traffic accidents have an adverse and significant impact on society. As these substantial economic losses arise from the cost of health services provided for injured people or productivity losses due to death or disability [2]. For instance, in the U.S., an annual estimation of USD 1 trillion accounts for an economic toll with a breakdown of an actual cost of USD 277 billion and USD 577 billion associated with productivity loss and a decrease in the life standards of injured, disabled, or killed people and their families [3]. Consequently, innovative and industrial automotive networking solutions have emerged out of necessity to address many safety and non-safety issues. These solutions included the introduction of connectivity into vehicles named connected vehicles (CVs), which, as a result, has been reshaping the transportation industry. These intelligent vehicles exchange traffic data among each other and with their surroundings to provide a better vehicle awareness and hence reduce road fatalities and traffic jams and provide a safe and convenient driving experience [4].

Generally speaking, CVs create new data-rich environments that enable many applications and services to make our roads safer, less congested, and more eco-friendly, while making our rides more enjoyable and productive. These applications include, but are not limited to, connected in-vehicle infotainment (IVI) systems, real-time navigation and routing, traffic information, safety warnings, accident avoidance, advanced driver-assistance systems (ADAS), automated driving systems (ADS), remote diagnostics, prognostics, repair or teleoperation in case of mal-functioning ADS, data monetization, and fleet management.

With the introduction of CVs to the automotive market, the aforementioned benefits and others are leveraged. Additionally, the wide deployment of sensor technologies within modern vehicles that aid in the design and development of various applications for traffic management, safety, and non-safety such as infotainment applications have greatly enhanced the driving experience. Even with the huge advancements in sensor technologies on transportation systems, there are still significant challenges. For instance, smart parking and reverse warning applications suffer from limitations in the fields of view beside sensor accuracy and robustness issues, since these applications depend on proximity and ultrasonic and electromagnetic sensors that are greatly affected by temperature and humidity conditions. Additionally, a lack of common recognized standards between various brands when integrating sensors with other components is another challenge that needs to be considered [5]. Therefore, the need to have advanced and efficient sensing and networking capabilities will help CVs reach their optimum potential, as gathering and collecting accurate data about their surrounding environment is a significant aspect in the connected vehicle technologies [6].

The CV global market dominance can be classified regionally. Geographically speaking, the largest share of the global CV market is currently dominated by the North American market due to their advanced and well-established networking infrastructure. This is followed by the European market, which comes second in place but is soon expected to be surpassed by the fast-growing CV market of Asia Pacific by the end of 2027. These are followed by the Middle East, Africa, and Central and South America. The CV global market is an increasingly highly competitive market due to industrial contributions and fast paced dominance by major market players, such as General Motors (GM), Volkswagen, Google, Tesla, Volvo, Mercedes, Bosch, IBM, Intel, Qualcomm, Huawei, and Vodafone [7].

Statistics and forecasting values reflect CVs’ promising future. IHS Markit predicts an expected worldwide sale of 72.5 million sold units by 2023. According to Statista [8], the revenue in the connected cars segment amounted to USD 21,811 million in 2019. Hence, they predicted an overall market growth from USD 46 billion to USD 140 billion. Moreover, based on the Juniper report [9], CVs are expected to reach around USD 775 million in the year 2023.

Everyday technological advancements bring CVs one step closer with enabling technologies and widespread adoption. We have noticed a growing interest from all stakeholders to make CVs a reality. Therefore, in recent years, CVs have attracted researchers to explore the enabling technologies, challenges, opportunities and to study the different aspects of the CV technologies. Lu et al. [10] conduct a focused review on the potential challenges and alternatives to achieve robust and reliable wireless communication technologies that enable V2X connectivity. The authors discuss the challenges that arise based on wired technologies for intra-vehicle communication, lower-layer-level (physical and medium access control) issues of dedicated short range protocols and Internet connectivity for inter-vehicle communication. Siegel et al. [11] investigate the design aspects of CV technologies and applications and review outstanding challenges in terms of privacy, scalability, and extensibility. The authors in [12] provide a precise overview on the features and functionalities of CV services along with the hardware and software technical challenges. In [13], the authors discuss the need for viable safety-based verification systems and point out the main three-fold aspects (modeling, analysis, and blueprints) associated with the control of the longitudinal and handling dynamics of self-driving vehicles. They further introduce the social and economic effects and provide suggestions on enhancements for traffic dynamics based on the adoption of connected and autonomous (CAV) vehicles. Although these studies focus on different aspects of CVs, we did not find a comprehensive review of CVs that provides a deep understanding of the enabling technologies, open challenges, and potential opportunities. This study provides a holistic review on the recent advancements of CVs technologies to comprehend the fast pace of the technological development in this field. Our contributions in this study are summarized as follows:

- Provided a comprehensive review with technical insights on the current standardization efforts of the key enabling communication technologies including Wi-Fi 6 and 5G.
- Discussed the challenges in regards to cooperative computation, privacy/security, and over-the-air updates facing CV technology and pointed out open research issues that need attention by the research community.
- Presented safety and non-safety applications and directed the reader’s attention to some unnoticed innovative applications’ research scope.
- Drew the boundaries of the opportunities that the CV technology brings to our life.

This article is organized as follows. Section 2 highlights the current enabling technologies that are suitable and most convenient for meeting CV technology requirements. Section 3 describes CV applications ranging from safety to commercial applications with an emphasis on applications that will have great potential in the CV environment. Recommendations upon fundamental challenges are presented in Section 4. Section 5 provides suggested opportunities to enhance and develop CV technologies. Lastly, conclusions are summarized in Section 6.

## 2. Enabling Technologies

The increasing demand for an “always connected” paradigm is pushing automakers to find more innovative software and electronics design that meet connectivity needs [11,14]. The automotive connectivity market share will continue rising up from the current 50% of CVs in retail to 95% worldwide sales in 2030 as estimated by McKinsey & Company [15]. Almost half of the sold CVs will include intermediate and advanced connectivity levels. The wide adoption of connectivity in the automotive industry will be considered significant when utilizing vehicle data to enhance safety, achieve cost optimization, and generate profit (up to an annual average of USD 310 income and USD 180 savings per vehicle in 2030). Monetizing connectivity to provide on-demand and customized connected features including subscribed in-vehicle hotspot and regular service check-ups based on the driver’s usage are receiving significant attention by the automotive industries [16].

The integration of connectivity into CVs will require underlying technologies to enable its deployment, functionality, and reliability within the intelligent transportation system (ITS) [17]. These enabling technologies will also facilitate the transmission and exchange of real-time data with high reliability as well as ensure smooth integration between smartphone devices and in-vehicle communication networks [14]. Minimal power requirements, cost, and scalability are critical factors that affect these technologies [11], especially in the case of electric vehicles (EVs). This section focuses on the enabling technologies for CVs and their current status to date.

Many efforts have been carried out by researchers to enable wireless vehicular communication in dense, dynamic, and harsh weather environments [11,18]. Wireless communication technologies currently used in connected vehicles mainly include dedicated short-range communications (DSRC), Wi-Fi 6, and cellular-vehicle-to-everything (C-V2X) communication. More ongoing research is exploring whether combining different communication technologies would result in a better connectivity for future CVs.

### 2.1. DSRC

**Figure 1:**
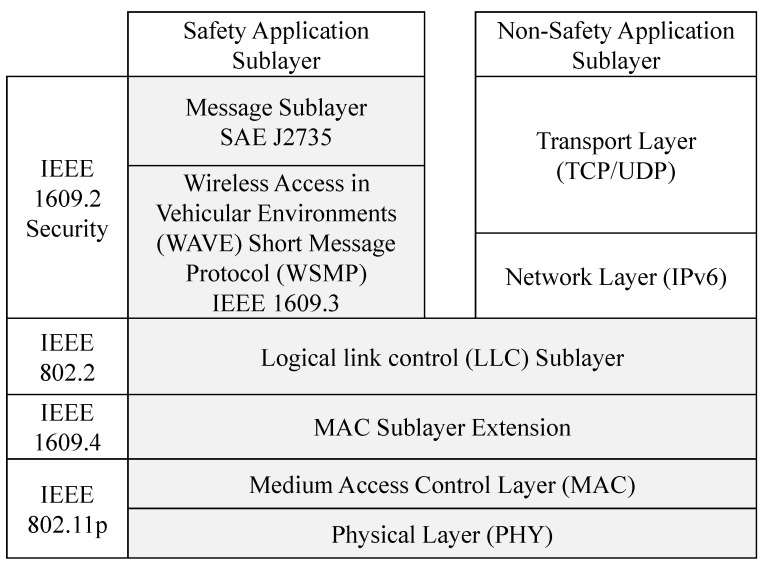
WAVE/DSRC communication’s layered architecture [22].

For over a decade, DSRC was one of the key enabling wireless communication technologies standards designed specifically for vehicular communication [10,19]. The IEEE standard for wireless access in vehicular environments (WAVE) presents the fundamental specifications of DSRC [20,21]. WAVE incorporates IEEE 802.11p, which deals with the medium-access control (MAC) layer and which tackles design challenges at the physical (PHY) layer of DSRC as illustrated in Figure 1.

The need for reliable and efficient wireless communication technology to support many of the rising innovative vehicular applications has resulted in further enhancements and developments in the current DSRC technology.

In spite of these efforts, in November 2020, the FCC has ruled on new regulations to reallocate the 5.850 to 5.925 GHz (i.e., 5.9 GHz spectrum band) dedicated for DSRC standard operations since 1999. The spectrum reallocation announced by the FCC comes as a drastic measure towards the slow deployment of the DSRC technology. The existing 75 MHz safety spectrum will be subdivided into two bandwidth allocations. The upper band of 30 MHz ranging from 5.850 to 5.925 GHz will no longer support DSRC and will be instead reassigned to advance and speed up the deployment of cellular-vehicle-to-everything (C-V2X) technology. The remaining 45 MHz of the bandwidth will be taken away and reserved for unlicensed wireless usage, presumably Wi-Fi [23].

In the automotive industrial field, there has been minimal adoption of the DSRC-based technology in any of the past original equipment manufacturers’ (OEMs) lineup production models. GM was the only automotive brand to adopt the DSRC technology in their Cadillac lineup production with only 50,000 DSRC-equipped vehicles rolled out. Although some auto manufacturers consider DSRC obsolete, LTE wireless communication technologies today cannot provide support for crash-imminent vehicle safety applications due to their high latency. 5G promises to handle this limitation, but it may take a while to be widely deployed and also need better training and understanding. Further concerns and skepticism are raised in regards to spectrum overloading and cross-interference that might occur from other radio-based devices utilizing adjacent frequencies to the 30 MHz spectrum where C-V2X technology is expected to operate. Even with the lack of research studies towards the aforementioned issues, the momentum shift towards the C-V2X technology is accelerating. Even though Toyota was one of the first automotive companies to support DSRC and integrate it in their 2021 production lineup, this plan has been recently put on hold to reevaluate their deployment environment. GM is considering cellular technology but has already integrated DSRC capabilities in the Cadillac CT6 and super cruise level 3 autonomous vehicles. Nissan is on the neutral side and is studying the potential and drawbacks of each technology; they are open to the deployment of any technology depending on the demand. Auto manufacturers have transitioned from the phase of the wait-and-see approach to the adoption of C-V2X technology in their lineups. Ford has already announced their C-V2X technology deployment in their production line for 2022 [23,24].

In the same year, SAE International announced a new publication of the SAE J3216 standard, which primarily focuses on cooperative driving automation through the integration of both connectivity and automation [25].

### 2.2. Wi-Fi 6

Wi-Fi 6 or IEEE 802.11ax is the latest wave of Wi-Fi technology. ABI Research predicts widespread adoption and domination of WiFi 6 chipsets in the automotive industry. These predictions imply that almost half of the Wi-Fi chipsets will be Wi-Fi 6 in 2023 and will subsequently reach a market domination of 70% in 2024. Other predictions claim that Wi-Fi 6 will be the in-vehicle wireless interface technology for CVs as it holds many benefits over previous Wi-Fi versions. Due to higher spectral efficiency/channel capacity, broader outdoor coverage, and flexibility, more customers can operate on the same access point and use cases demanding higher bandwidth, such as high-quality streaming videos, which can be supported by Wi-Fi 6. Overhead reduction due to small-sized traffic data is also endorsed. Wi-Fi 6 also degrades signal congestion by incorporating orthogonal frequency division multiple access (OFDMA) (for multiple users with differing bandwidth requirements) and a basic service set, which is referred to as BSS (for signal identification from heterogeneous radio devices). The aforementioned benefits of Wi-Fi 6 technology will meet the demand of the exponential growth of automotive safety/convenience applications and use cases by allowing a seamless wireless connectivity inside/outside the vehicle and an enhanced quality of experience (QoX) for users [26,27]. Even with the aforementioned benefits, Wi-Fi 6 will have a smaller coverage range in comparison to 5G-cellular technology.

### 2.3. 5G-Cellular Technology

Cellular technology such as the fifth generation (5G) is another strong competing candidate to achieve efficient and reliable wireless communication to support CV applications. The network capacity in cellular technology is high, which provides higher bandwidth and which can have the ability to support a variety of data-based applications. Cellular technology covers a wide coverage range, which diminishes frequent horizontal handovers. In this case, vehicles can stay connected to the base station for a large amount of time, in contrast to the small duration of time between vehicle and RSU, where the communication link remains valid [28]. Moreover, it precisely focuses on the performance measures related to achieving ultra-low latency, improving reliability, decreasing the power usage, and maximizing network throughput per session [29].

The rise of 5G mobile cellular networks as a new innovative technology opens up the potential to enable fully mobile and interconnected systems. The Third Generation Partnership Project (3GPP) release 15 [30] and Technical Specifications (TS) 23.501 [31] provide a reference framework for a collective number of requirements and functionalities needed to advance and establish 5G networks [32]. Moreover, 5G automotive association (5GAA) is an organization, where major automotive and telecommunication corporations including BMW, Audi, Ericsson, Intel, Nokia, and many others come together to advance, employ, and provide mobility and transit solutions for cellular-based V2X communication [32,33]. 5G networks will depend on long-term evolution-advanced (LTE-A), millimeter WAVE (mm WAVE), Wi-Fi, and Wireless Gigabits (WiGig), which are recent radio-supporting technologies, operating from ultra-density-based cells to device to device (D2D) [32]. The design of LTE-A meets the safety- and non-safety-based application specifications including high mobility, wide coverage range, improved quality of service (QoS), and multicast communication. LTE-A downlink and uplink access technologies will utilize OFDMA and single-carrier frequency division multiple-access (SC-FDMA) techniques allowing for scheduling based on frequency–time resources to be achieved with high resilience and effectiveness. Spectral competence can also be achieved in a highly dynamic environment depending on advanced multiple input multiple output (MIMO) potentials. Even with the previously mentioned benefits, LTE-A still suffers from latency issues where the maximum achievable latency is around 80 ms, which is not convenient for applications that require a maximum latency of 10 ms [29]. Whereas, mmWave incorporates beamforming technology that allows higher data rates, in addition to its bandwidth of 30–300 GHz that significantly enhances system operations [29,33]. The Doppler effect at mmWave frequencies can be controlled in a highly dynamic environment due to the presence of directive transmissions and narrow beam-based antennas [33].

The fundamental technological design of 5G is dependent on the automation of the resources of the network that utilizes network slicing (NS), which is dependent on network function virtualization (NFW) and software-defined networks (SDN). Issues such as improper connectivity and the packet loss rate can be solved through the integration of SDN and edge computing. Vehicular networks based on SDN can benefit from such technology such as improving resource utilization [34].

(1) Challenges of 5G-Cellular Technology: With the high expectations that 5G will meet the dynamic change of vehicular networks, high mobility endorsed by vehicles and the huge volume of data transmission involved within the CV technology [32], 5G telecommunication is still facing several challenges, which are further discussed below:

- **Service and Business Models.** The increasing number of connected IoT devices to the internet through 5G necessitates the requirement for new business and service paradigms. The 5G network technology is deemed ideal for connecting IoT devices, and hence forming a separate subscription for every device is considered insignificant. This derives the investments to improve and enhance the infrastructure network to be capable of delivering QoS services at the edge. Moreover, the impact of 5G bandwidth on the backhaul networks and the frequent dynamic of the mobile devices impose a great challenge for delivering services in regards to virtualization and edge computing [35].
- **Centralized Architecture of Cellular Networks.** The centralized-based architecture of the cellular networks will pose a great challenge in correspondence to CV environments. By design, transmitted information by vehicles have to be initially sent to the base station (BS), and hence it extends the latency for message delivery. This imposes drawbacks in safety time-critical applications, where these applications require ultra-low latency. For example, in a unicast mode situation, a vehicle transmits safety-critical information to the cellular BS, and this message is then either broadcast to all the vehicles within the BS’s cell range or to the pertinent vehicles only. In these two situations, the downlink channel becomes traffic jammed even when there are a considerable number of vehicles [28,36]. Proposed solutions for broadcasting safety messages include multimedia broadcast and multicast services (MBMS) along with the evolved multimedia broadcast and multicast services (eMBMS), which are incorporated in the 3GPPP standard. In broadcasting situations, the safety data is broadcast by BS to all vehicles in its cellular range. In this manner, vehicles decide on the relevancy of the received safety data whether to use this information or discard it. Thus, vehicles may perform unnecessary computation. The utilization of multicast services, where data is delivered to a multicast group of vehicles, is considered to be one of the proposed solutions for this issue [28].
- **Interference at Low-level Altitude.** In CV environments, vehicles should be able to discover and communicate with nearby vehicles occasionally. A significant specification of 5G-enabled telecommunication is the availability of the proximity service (ProSe) [37,38]. The main purpose of ProSe is to enable vehicles to have a better perception by discovering devices and services based on the position and geographical location information [21,38]. ProSe is considered critical for many applications and communication opportunities within a specified range. Moreover, it provides communication and discovery in an ad-hoc manner, which is considered most convenient for safety-critical applications. However, the paradigm shift in ProSe gives rise to radio propagation among vehicles that is caused by high buildings, towers, and many other obstructions in urban canyons and metropolitan areas, for example, which lead to a high level of interference [38].

(2) Current Status of 5G-Cellular Technology: Current cellular technologies are able to provide some of the requirements needed by CVs. For example, LTE-CAT-M and narrow-band Internet of Things (NB-IoT) are considerably reliable and adopt minimum power sensor telecommunication technologies. Yet, broadly speaking, cellular technologies fall short to meet many of the demanding requirements needed by V2X applications such as high speed, minimal latency, complicated vehicle maneuvering, and many others. Many parties including some of the leading automotive, telecommunication industries, and governments are driven towards 5G to leverage its benefits into ITS. In an effort to support 5G technology, a board of leading companies are aiding to advance a comprehensive 5G system architecture to leverage an optimized end-to-end V2X connectivity. A project named 5GCAR financed by the European Commission, which incorporates 14 European companies such as Bosch, Orange, Volvo, PSA group, and many others, has been launched in support of these efforts. In June 2019, the first use case ”Lane-merge coordination” was exhibited in France by the consortium to demonstrate the optimization of merging vehicles entering the highway. The vehicles are able to share their current state data with a centralized maneuver planning system and hence upon these data sharing, recommendations regarding acceleration, deceleration, and lane changing is provided to the CVs. Another use case demonstrated by 5GCAR is “cooperative perception” to show communications between vehicles and vulnerable road users (VRUs) [39,40].

The rollout of 5G NR networks is considered indispensable to endorse the aforementioned use cases and many others. In this context, the deployment of 5G NR would be predominately classified into two main operation modes, namely, non-standalone (NSA) and standalone (SA). The NSA mode will include the deployment of the preliminary stages of 5G NR through the assistance of the current 4G infrastructure in order to speed up the 5G network’s implementation. The NSA operation mode will hold a number of advantages due to the interconnection between 4G and 5G networks. This includes a reduction in the 5G network deployment time and support for dual 4G/5G operated devices. The core architectural components of the NSA mode are LTE BS (eNB), LTE evolved packet core (EPC), 5G BS (gNBs), and 5G core (5GC) networks. This is in addition to incorporating multi-radio access technologies (RATs) and dual-supported connectivity for customers. On the other hand, the SA operation mode incorporates the implementation of the entire version of 5G NR. 5G NR operating in the SA mode will leverage complete end-to-end 5GC-based networks and architecture that can provide use cases with ultra-low latency and massive capacity. Thereby, the interconnection between gNBs and 5GC networks will exploit 5G cells for information transfer in user/control planes. 5G NR networks in the SA operating mode connected with cloud native 5G core will improve ultra-reliable low-latency communication (URLLC) and support a wide-range of use cases and advanced NS-based functionalities and services. Ease of implementation, improved dadio access technologies (RAT), and better performance are additional advantages offered in the SA operating mode. However, one of the challenges that pertain to the SA mode is the wide coverage adoption of 5G [41,42].

New radio (NR) V2X is the latest advancement that 3GPP is in the process of developing and standardizing for release 16. It is built on top of 5G NR, which has been standardized in release 15. NR V2X has great potential with respect to V2X applications that demand higher QoS requirements in comparison to applications supported by cellular-V2X. Some of these applications require ultra-low latency that can reach up to 3 ms with a reliability of 99.999% [43]. These advancements in 5G-cellular technology are expected to further leverage the potential benefits of the CVs by enabling various add-on services that can meet the QoX and QoS requirements. QoX is the degree of delight or annoyance of the end-user resulting from the fulfillment of his or her expectations with respect to the utility and/or enjoyment of the technology with respect to the user’s personality and current state. The QoX perception process is known to be dependent on a triad of influential factors, namely, the vehicle being used and its specific features and conditions, the context in which it is being used, and the subjective (or human) personality and mental state of the user. Context is any information that can be used to characterize the situation [44]. This contextual information answers situation-related questions such as who, what, when, and where in order to characterize the environment, the vehicle, and the users in terms of location, identity, activity, time, and status. Context can also include communication medium aspects such as bandwidth, latency, and topology. Taking this contextual information into consideration can help in envisioning new services like triggering promotions as part of loyalty programs based on the location or adapting the connectivity aspects based on the availability of bandwidth or other QoS parameters. Examples of this adaptation may include establishing an ad-hoc V2V communication relay in case of losing the communication with the infrastructure or if the infrastructure we want to reach is outside the communication range of the vehicle. In-vehicle infotainment services, such as delivery of high-definition videos/maps and real-time interactive games, exemplify the new means of convenience that can be provided by the new cellular-V2X technology [45].

To reach a reliable, established and secured seamless connectivity for V2X applications/services, it is crucial to provide feasible connectivity solutions that can be integrated with the built-in V2X products installed in CVs [28,45]. In this context, a step forward towards 5G connectivity is the latest Qualcomm Snapdragon 5G platform [46] that provides various functionalities to empower CVs. The integration of C-V2X direct-based communication to enhance road safety and 5G dual SIM dual active (DSDA) technology that gives the chance for drivers and vehicles to select and operate on different network operators independently are examples of the various features provided by Qualcomm Snapdragon 5G platform [47]. Other companies, such as Pirelli, have developed smart tires that utilize the 5G cellular networks to relay hazardous road-surface data to the driver and nearby vehicles. In 2019, a field test was conducted to validate the efficiency of these smart 5G-connected tires in detecting the presence of aquaplaning hazard and alerting nearby vehicles [48].

Table 1 provides a focused comparison between DSRC and 5G-cellular technology, in terms of latency, communication range, benefits, and challenges.

### 2.4. Hybrid Architecture

In the aforementioned subsections, the benefits and drawbacks of DSRC and cellular technology in regards to the vehicular environment were discussed. Recent efforts [54,55,56] have been directed towards the integration of both enabling technologies to develop and improve vehicular wireless communication by exploiting the complementary aspects of DSRC and cellular technologies. This is because V2X applications and services demand effective and efficient usage of diverse access wireless communication technologies [57].

**Figure 2:**
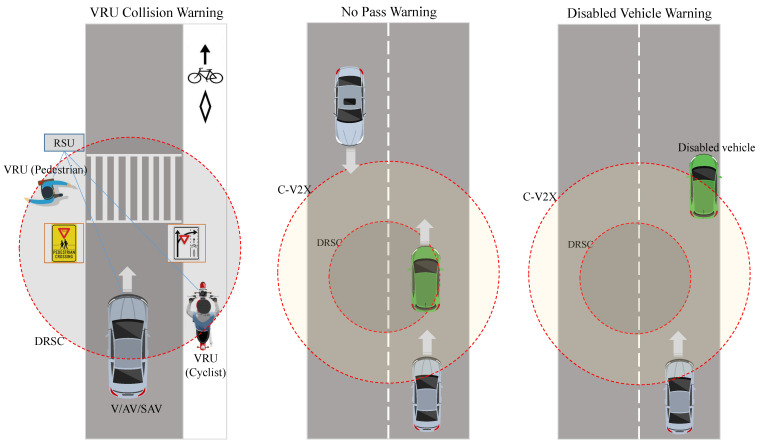
VRU Collision warning, no-pass warning, and disabled-vehicle warning.

Inter-working between DSRC and cellular technology, which forms a hybrid solution, is considered promising, as it utilizes the benefits of both enabling technologies. For instance, in situations where transmitted data between vehicles are shattered and V2V multi-hop communication fails, cellular technologies may perform as a backup solution to relay transmitted information. Moreover, vehicles may reconnect to the Internet access network of cellular technology in case of Internet connection loss with the RSUs. Figure 2 illustrates examples of safety applications where the integration between DSRC and cellular technology will be beneficial.

Additionally, the integration between DSRC and cellular technology can also be utilized to ease effective clustering techniques. In clustering techniques, nearby vehicles are grouped into clusters, where the sustainability and handling of network resources of each cluster is achieved by a cluster head (CH). Moreover, remaining vehicles within the cluster are referred to as cluster members (CMs) and may be part of several adjacent clusters. CH may choose some vehicles to perform as a gateway to ease the communication between neighboring clusters. In a cluster-based manner, CHs and gateways form a dynamic hierarchy that allows two-way data transmission from and to cellular networks; for instance, a multimedia file is downloaded by CH from a cellular network and then DSRC technology is utilized to disseminate it to its CMs in the cluster [28]. The main benefits of clustering techniques are efficiently decreasing the size of the network and the delay in each cluster. However, the rapid change in the mobility of vehicles affects the cluster steadiness as a result of its effect on the splitting and merging of the cluster, which imposes considerable cluster control overheads on the network and hence necessitating the need for effective and minimal overhead techniques. Clustering stability is a challenge that should be considered when implementing a hybrid architecture of DSRC-cellular networks that depend on a cluster approach [28,58].

Another important aspect of the hybrid architecture of DSRC and cellular technology is the strategic paradigm used for network selection between both enabling technologies. One of the challenges that should be carefully considered when designing the network selection paradigm is the driver’s preference to sustain their current network without swapping to another network for trivial increase in performance and quality. Moreover, in a transition stage where not all vehicles are equipped with devices that allow their interconnection between DSRC and cellular technology, fairness concerns may emerge, especially in cases where decisions for network selection and handover are taken merely by each vehicle in accordance to its own preference [28].

The hybrid architecture of DSRC and cellular technology is foreseen by ABI research [59] to be more cost effective and easier to implement and design. DSRC–cellular hybrid architecture is considered to leverage promising prospects, but it is important to consider challenges at an earlier stage of its deployment.

## 3. Applications

The connected vehicle applications are designed and developed to address many of the current modern transportation issues [60]. In regards to the performance and implementation cost of these applications, they greatly depend on the design decisions such as real-time input sensor data, modes of communication used, location of the computation process, and secure data transmission [11,60]. The wide umbrella of connectivity is vehicle-to-everything (V2X), which secures the sharing of information seamlessly between the vehicle and everything in the right format at the right time. There are several potential applications for various forms of connectivity within the V2X paradigm, such as the following:

- **Vehicle to occupant (driver/passenger) (V2O).** Enabling features like BLE/UWB-enabled phone-as-a-key; in-vehicle connectivity services for work, play, and commerce; driving error recognition and prediction; and QoX.
- **Vehicle to vulnerable road users (V2VRU).** VRUs include pedestrians/jaywalkers and cyclists as well as motor-cyclists and persons with disabilities or reduced mobility and orientation. V2VRU can be an enabler for VRU detection, crossing intent and motion behavior recognition, and pre-crash warning.
- **Vehicle to vehicle (V2V).** Applications incldue post-crash warning, pre-crash warning, lane-change warning, cooperative collision warning, cooperative adaptive cruise control, visibility enhancement, wrong-way driver warning, intersection movement assistance, blind-spot warning, cooperative forward-collision warning, vehicle-based road-condition warning, communication relaying in case of emergency, smart cargo companions, last-mile delivery systems, fleet management systems, and self-organized autonomous vehicles.
- **Vehicle to environment (V2E)** includes road-condition monitoring, traffic-sign and light recognition, driving risk prediction, and perimeter monitoring systems.
- **Vehicle to infrastructure (V2I).** V2I is used for road-condition warning, SOS services, work-zone warning, do-not-pass warning, emergency vehicle signal preemption, intersection collision warning, in-vehicle amber alert, remote diagnostics and repair, pedestrian crossing information, red-light warning, pedestrian detection and warning, bicycle detection and warning, no left-hand turn warning, traffic condition monitoring, weather conditions, traffic light management systems, interaction management systems, parking management systems, and teleoperation in case of malfunctioning self-driving cars. For example, OnStar services provide automatic collision notification, enhanced roadside assistance, SOS emergency assistance, automatic diagnostic trouble code notifications, monthly vehicle health reports, service links, maintenance reminders, driving information, and on-demand diagnostics.
- **Vehicle to network (V2N).** V2N is used for disabled roadside vehicle warning, security credential management systems, multi-model mobility systems, dynamic on-demand mobility systems and services, cloud-based crowd-sensing services, real-time traffic monitoring, and for bringing attractive consumer experiences into the cabin to foster brand loyalty.

In this section, we shed light on the safety and non-safety applications. However, mobility [61,62] and environment applications [63,64] also utilize the CV paradigm to provide many benefits including smooth traffic flow and eco-friendly road-trip decisions.

### 3.1. Safety Applications

The advancements in safety systems, such as active and passive systems, have been growing rapidly to mitigate many fatal traffic accidents and to help drivers and passengers survive. Active safety systems assist drivers in avoiding accidents through in-advance audible and visual alarms or by assisting drivers in managing their vehicles, such as through anti-lock braking systems. These systems are considered preventive systems, which help drivers avoid accidents before they occur [65,66]. Whereas, passive safety systems help minimize accidents’ impacts on the drivers and passengers in case of accidents; these include seat belts and air bags [66]. The integration of wireless communication in the automotive industry has led to many innovative active-system wireless-based applications among vehicles and between vehicles and infrastructures. On that account, these applications or cooperative safety driving applications provide traffic-related data to drivers beyond their perception, and, hence, they mitigate traffic accidents and reduce congestion [65].

CV applications demand low latency, efficient, fast, and reliable broadcasting protocols for delivering messages in single-hop and multi-hop communications. However, some of the common issues associated with dissemination algorithms include the hidden node problem, the unavailability of feedback. and the high rate of collisions in the broadcast messages [67]. Several forms of message broadcasting can take place in vehicular environments. This includes beacon messages that send a vehicle’s status and situational data in high frequency and small packets in a periodic manner. Even though beacon messages are considerably important in cooperative awareness applications, it suffers from some pitfalls such as latency, network flooding, and a lack of location preciseness [11]. Whereas, event-driven messages transmit critical event-based information, for instance, in cases where safety critical events should be noted; therefore, these messages will require minimum delay and a lower collision probability rate [11,68].

**Figure 3:**
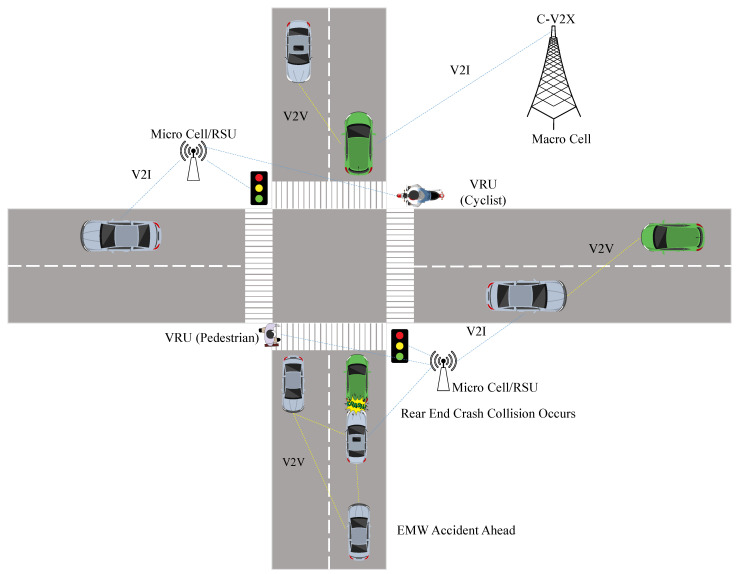
Post-crash safety warning scenario.

Safety-related data such as basic safety messages (BSMs) help drivers to have a full real-time awareness of the surrounding environment, since they constantly share their speed, acceleration, and direction and other relevant safety data. Another form of safety traffic data is event-based data, as shown in Figure 3, where a vehicle ahead of the way is able to notify other vehicles about incidents that lie ahead or an alarming situation, such as emergency electronic brake light ahead warning or forward collision avoidance [69].

Safety applications are time-critical applications, which require real-time data to be delivered with low latency, reliability, and a high transmission speed. However, to enhance latency and performance, challenges with respect to data management and analysis should be addressed. Even with the high cloud scalability and its low cost, solutions such as, increasing the computational power are not feasible in terms of data management challenges [11]. Moreover, safety applications will require thorough analysis in terms of safety evaluation and modeling [70,71,72]. In the following subsections, some of the most popular CV safety applications are discussed.

#### 3.1.1. Red-Light-Violation Warning (RLVW)

In this application, a micro-cell/RSU sends both signal timing and the geometry of the intersection in signal-phase–timing (SPAT) and MAP messages, respectively. The vehicle uses this information along with its speed and acceleration to determine if it is likely to violate the red signal or not [73]. For example, a warning is issued to the driver if the vehicle is approaching an intersection at the start of the yellow signal and the time needed to cross the intersection is longer than the duration of the yellow signal [74].

Various studies have been carried out to enhance and improve the RLVW application performance. Prior knowledge of red-light-running violations can help in detecting such incidents and in notifying nearby vehicles in advance to take proper actions. In [75], the authors came up with a system model that can predict red-light-running (RLR) to assist in diminishing the fatal accidents caused by such an action. Their proposed model embraces the use of time-of-flight (ToF)-based light detection and ranging (LIDAR) sensors for speed calculations along with a prediction algorithm that is dependent on computing the stop sight distance (SSD) formula to determine the braking distance with respect to the speed. LIDAR positioned at the side and front of the vehicle is able to determine and measure the speed of vehicles approaching the intersection and hence is able to anticipate the RLR violation that the vehicle might experience. The traditional TOF LIDAR sensor technique is widely adopted and is a popular approach for speed/distance estimation due to its simplicity, precision, and sensitivity in comparison to other proximity-based sensing techniques [76,77]. However, this technique can still suffer from complex design during detection due to high amplification factors and frequency rates [77]. Chen et al. [78] describe a probabilistic stop-or-go prediction framework that depends on Bayesian networks (BN). The authors broaden the deterministic output into a probabilistic output. The BN framework is adjusted and assessed through the streaming trajectories of data gathered by the radar sensors that are employed in the signalized intersection. Many factors are incorporated in their model including the dynamic movement of the vehicles such as speed and accelerations, in addition to considering car-following behavior. Their probabilistic RLR BN prediction framework provides a high accuracy level, and the model’s performance is enhanced with feature interpretation. In [74], the impact of voice warnings on the brake response time (RT) to RLR violations in the presence of collision avoidance process was analyzed. The analysis was conducted and tested on a driving simulator. To test the effectiveness of their system warning status, lead time and content were all taken into consideration. Their experiment demonstrates that using voice warnings was able to reduce the RT; hence, the collision occurrence rate was also reduced. Both early and late warnings were able to reduce the RT and the collision occurrence rate, with better results achieved with early warnings. The authors point out that their results can help designers of the RLR collision warning systems to improve the effectiveness of those systems [74]. In their experimental research study, the authors incorporated several demographic factors, such as driver’s age, gender, and experience. However, the driver’s style and uncertain behavior can also have a significant impact on the braking response time and the RLR violations, which may lead to unsafe signalized intersections [79].

#### 3.1.2. Emergency Electronic Brake Light Warning (EEBL)

In this application, drivers are alerted of an emergency hard braking event performed by vehicles in front of them to be able to take the proper action to avoid an accident. The emergency hard braking event flag means that an event flag is incorporated within the data elements. Event flags’ data elements are not included within the BSMs except if one of those flags are set to one [80]. EEBL application is considerably useful in situations where poor visibility exists, for instance, harsh weather conditions such as heavy rain or when the driver’s sight is blocked by other vehicles [81,82].

Several studies have been directed to enhance and develop the EEBL application performance. For instance, authors in [83] present a comparison between two approaches carried out to evaluate the significance of electronic brake reports in EEBL systems. The first technique was an analytical equation that depended on the minimum safety gap scheme. The second method was a machine learning tactic that was designed and proposed by the authors. In this context, reports regarding decelerated vehicles were being broadcast by their application to inform and alert drivers in need to conduct emergency braking. Based on the relevance of this report to the driver, vehicles decide whether to notify drivers or not. The lane and direction of the data were both common features to determine the relevance; however, false-positive warnings can occur, which may lead drivers to disregard these warnings or switch off the system, hence ignoring any safety benefits of the application. According to the analysis, the machine learning model outperformed the analytical technique in decreasing the number of false positive warnings by learning from the post actions taken by the driver upon obtaining the report. Both methodologies were compared using the MITSIM simulation software with a variety of traffic and communication specifications [83]. In another study, Payre et al. [84] present the design and assessment of in-vehicle signs shown on the smartphone to applications including EEBL, electronic-vehicle warning (EVW), traffic-condition warning, and road-work warning. They evaluated their design using quantitative and qualitative techniques. A total of 44 participants were provided with 11 design options, and their display included both non-legend and legend designs. The results showed that EEBL and EVW signs based on the legend design were better understood and less vague, in comparison to non-legend-based signs. Displaying a legend design underneath the signs to alert drivers of any potential emergency braking that might occur ahead was effective in situations where poor visibility conditions exist. Hence, an improvement for safety-critical incidents can be achieved.

#### 3.1.3. Curve-Speed Warning (CSW)

CSW applications are developed to inform drivers of any approaching curves, in case their current speed is considerably high and unsafe to pass through the curve. The application incorporates data provided by the infrastructure along with the vehicle sensor data to provide a recommended safe speed. The recommended speed depends on the geometrical shape of the curve, road and weather conditions in real-time, vehicles dynamics, and stability telematics information [85,86].

Serna et al. [87] have shed light on the lack of road geometry considerations in intelligent speed adaptation systems. Based only on fixed speed limits, drivers are only warned when they go over the suggested speed. The authors demonstrated a dynamic speed adaptation methodology that provides an ideal speed taking into consideration the curvature of the road. They also performed curve analysis extraction using an algorithm that utilizes the GPS data off-line identifiers, which indicate immense curvature segments and which provide speed estimation for each curvature.Their proposed model makes use of the GPS data based on real-time kinematics GPS to sense maximum speeds and to identify road curvature sharpness to guarantee a smooth shift between the existing speeds. The model has been put for testing through experimental simulations, which indicated a reduction in lateral errors on sharp curves [87]. These results were conducted using a simulation-based approach, and it would also be important if their findings were additionally validated using field test experiments to provide a more contextual analysis of the lateral errors. Moreover, the real-time kinematics GPS adopted in their study had high precision and centimeter-positional-accuracy levels, but it is still prone to residual atmospheric errors in long- and medium-distance ranges [88]. In [89], the authors discuss the potential of improving the effectiveness of CSW systems by adjusting to the driver’s behavior instead of adapting only to road and traffic conditions. A personalized adaptive curve speed warning (ACSW) system adapts to the driver’s response time and historical curve handling performance. On the other hand, the waiting time is modified according to the reward/punishment function that helps reinforce safer actions, while providing customized time alerts. The authors also conducted a comparison between ACSW and CSW and their findings, which suggest that the interactions of drivers with warning systems greatly depend on demographic factors, such as gender and age. Moreover, their results indicate that the driver’s near-curve speed changes significantly according to road attributes such as advisory curve speed and curve direction.

#### 3.1.4. Platooning

Cooperative vehicle/truck platooning is considered one of the most promising applications that has received significant attention from both automotive industries (e.g., Volvo, Daimler, and Scania) and academia. Vehicles/trucks in platoons are expected to maintain safe inter-vehicle distance and synced speed, routes, and braking through sensors/radar technology utilization, which is enabled by V2V communication when closely following a leader vehicle/truck. Platoons will benefit from significant reduction in fuel consumption, congestion, harmful gas emission, and aerodynamic drag. Reduction and improvements in aerodynamics drag will specifically have a remarkable impact on trucks, which are susceptible to higher wind resistance in comparison to other vehicles. Trucks in platoons are expected to save up to 15% of fuel consumption when auto-following their leader truck. Even with the aforementioned benefits, inter-platoon communication interference may arise among adjacent/opposite platoons, especially in intersections or crossings, where a number of platoons may coexist. Remote leader truck hacking, out-of-the loop drivers in tailgated trucks, abrupt software/hardware failures, real-world heterogeneous platoons, and platoon stability are all among the challenges of platooning application. One of the proposed solutions to tackle out-of-the-loop drivers is the deployment of a level 1/level 4 platooning system, where the lead truck will be operated by a human driver to combat any unexpected road conditions or adverse weather situations followed by tailgated level 4 automated trucks [90,91].

Furthermore, research studies have been dedicated to enhance both centralized (followers communicate with platoon leader) and decentralized (peer-to-peer platoon communication) platooning systems [92]. The authors in [93] propose platooning as a service framework that adopts a centralized mobile edge computing (MEC)-based approach to control the speed/acceleration of follower vehicles in the platoon. Uplink/downlink transmissions’ latency/packet loss probability impacts and electromechanic actuation lags of vehicles/trucks were investigated in the SUMO simulator. The proposed platooning system was tested on highways; however, other traffic flow scenarios, such as intersections should be considered. Other studies adopted a decentralized approach such as [94], where the authors proposed a distributed resource allocation (channel assignment/power allocation) algorithm, which was developed for C-V2X-based platoons. Optimization decisions based on a deep reinforcement learning algorithm are taken by the leader vehicle to allocate the optimized sub-band and power of transmitted signals to the auto-following vehicles. Reduction in signal overheads and near-centralized V2I sum-rate performance were achieved and validated using the simulation. Another interesting aspect is the communication latency within platoons of vehicles, which has been tackled in several research studies. For instance, the authors in [95] proposed an adjusted cooperative adaptive cruise control (CACC) framework for longitudinal safety and sensitivity analysis of the communication latency for vehicles’ platoons.

### 3.2. Non-Safety Applications

With the amount of data shared among vehicles, many non-safety applications and services can be developed in connected environments. Applications such as traffic management are aimed at decreasing traffic congestion and enhancing traffic flow. This is done by sharing traffic monitoring information that helps drivers reroute their destination, and traffic engineers optimize the traffic-light scheduling, thereby decreasing traffic congestion. Infotainment applications are another example that mostly provide location-based services and entertainment such as parking availability, hotels and restaurants locations, music streaming, and file sharing [65]. In the following subsections, we discuss some of the interesting non-safety applications that could be offered by CVs technologies.

#### 3.2.1. Infotainment Applications

In the connected vehicles era, drivers expect more from vehicles beyond safety. The increasing demand towards an *“always connected”* paradigm and the growing need for in-vehicle entertainment applications along with the capability of vehicles to connect to the Internet have paved the way for many innovative infotainment and multimedia applications including high-quality music and video streaming, interactive gaming, and file-downloading [10,12]. These applications require Internet connectivity. Since automakers integrate their own infotainment systems into their own car production lines, there exists a major difference between automakers as to whether these systems should have a built-in Internet connectivity or should use the drivers’ smartphones for Internet access. However, most automakers are in favour of in-vehicle Internet, and, hence, great efforts have been conducted to include a wireless modem within vehicles to provide Internet and device connectivity within the vehicle’s dashboard. This will allow the integration of in-vehicle sensor data with web information, which provides a convenient, safer, and more secure driving experience [12,96].

#### 3.2.2. Vehicle as a Mobile Service Provider

Other potential applications beyond entertainment can be provided by utilizing the sensing, communication, and computation capabilities within the CVs and hence enable CVs to act as a mobile service provider for many entities including car riders, nearby vehicles, and third-party recipients [97,98]. This includes providing services, such as data relaying, commercialization, and localization and data storage services [98].

In the data-relaying services, connected vehicles act as forward data relays that enable a source vehicle to communicate with out-of-range destination vehicles through intermediate vehicles. Data relaying can take place among vehicles and between vehicles and microcells/RSUs. In this context, data-relaying services can be used as a methodology to increase network coverage. Data relaying in connected vehicles can follow one of three communication forms that include geo-casting, unicasting, and information dissemination. In geo-casting, connected vehicles relay data to a group of destination vehicles that are geographically located in specific zones of interest, and then the data is sent back to the source vehicle, for example, traffic-related data that include road conditions in a specific location, such as gas stations, where vehicles send their real-time speed indicating the current traffic at this specific location. Unicast means that vehicles relay data to a single destination vehicle. Information dissemination refers to the flooding of data to all neighboring vehicles. This takes place in cases of road-hazard situations, such as pothole detection using in-vehicle sensors. This information can be disseminated to other vehicles to warn them of the harshness and location of this pothole. Moreover, advertisement agencies can make use of the mobility features of CVs to advertise to drivers/passengers’ special offers by hotels, restaurants, shopping malls, etc. Hence, commercialization is a paid service to the driver whose CV relays information about offers and advertisements while on the move [4,98].

### 3.3. Research Potential for Applications

The growing demand for more innovative CV applications have witnessed unprecedented and huge strides in recent years. In this section, we provide a precise look at some of the promising CV applications and direct the reader’s attention to some unnoticed research scopes.

#### 3.3.1. Data Monetization

Data monetization refers to turning collected data into a monetary value [99]. In this context, data takes a new role where businesses can productize these data to promote new services, hence increasing their revenue streams [100,101]. The monetization of the collected data from millions of CVs on the road promises to enable more innovative and compelling CV services [102,103]. Many automakers are seeking to leverage CV data for various innovative services. GM, for example, introduced a new service called “Marketplace,” where drivers can use the infotainment dashboard to place orders (e.g., coffee and gas reservations) or to book restaurant seats in advance, while on the move [104]. Fuel depot is another service provided by GM that assists drivers with low fuel tanks to find the nearest gas station and, based on this information, provides offers and discounts that might be available at nearby gas stations [105].

Automotive industries are racing to offer sophisticated and advanced in-vehicle services to improve the customer experience and to leverage data monetization opportunities. The Connected Car Customer Experience (C3X) framework proposed by McKinsey & Company [15] shows how CV customers’ in-vehicle experience can be monetized and serves as a framework to estimate the data-driven value-generation opportunities aligned with increased user connectivity. The C3X framework defines five levels of customer in-vehicle experience in CVs extending from a basic to a high level of vehicle connectivity. These levels are scaled up to reach the ultimate cognitive artificial intelligence advanced level where vehicles can predict and achieve unexpected driving situations. More detailed information on the C3X framework can be found in [15,16].

The high revenues expected to be generated from monetizing CV data are hampered by a number of challenges, one of which is promoting the added value associated with new connected services that require complex installation procedures and that might be faced with connectivity issues. The lack of a standard data monetization framework throughout the vehicle life cycle and struggling to model agile-based systems and non-scalable ecosystems are also other challenges [15]. Exploration of how to monetize data efficiently without privacy invasion is important for both OEMs and users. Moreover, it is important to consider cases where poor data quality exists and provide innovative solutions to improve data quality and integrity.

#### 3.3.2. Last-Mile Delivery Services

The last mile is defined in transportation planning and supply chain management as the movement of goods and people from a transportation hub to a final destination [106]. The growing public demand for online shopping has greatly increased the number of delivered packages by courier, express and parcel services. People tend to order online aiming at fast and more time-efficient delivery services. However, the direct relation between door-to-door delivery and the inefficiency of the last-mile delivery systems has potentially resulted in significant extra miles traveled by diesel trucks, specifically in residential areas. These trucks contribute to traffic congestion, air pollution, higher costs, and low quality-of-life conditions. It becomes even worse when customers are required to sign upon delivery [107,108]. Therefore, innovative solutions for last-mile delivery to decrease the negative environmental impact of the extra miles traveled are becoming a necessity. These solutions include, but are not limited to, cargo-bikes [109], semi- and fully autonomous last-mile delivery, delivery droids (bots), E-palette, postal delivery, driverless deliveries, and privately owned AV. As an example, cargo bikes include the Centaur Cargo Trike Xl and Armadillo Volvo’s electric cargo bikes [110]. According to McKinsey, semi- and fully autonomous last-mile delivery will reduce costs by approximately 10–40% [111].

The COVID-19 pandemic has taught us the lesson that our systems are not designed for large-scale emergencies. The high contagiousness/viral shedding of the novel coronavirus is one of the major causes of the outbreak. Social distancing is the main measure taken to reduce the spread of the virus through minimizing the contact between the people. However, frontline workers who treat patients or who deliver medicine or food for them, and even people at home who order essential goods like medication or groceries, are still at risk. Contactless last-mile delivery systems and services can help in avoiding physical contact between caregivers and patients or between delivery workers and the delivery recipients. These contactless last-mile delivery systems will benefit from the rapid proliferation of connected technologies and the recent advancements in semi- and autonomous delivery platforms to revolutionize the urban logistics and to provide safe and efficient delivery methods for medical supplies [112], medications [113], food, groceries, and other goods [114,115].

Increasing the efficiency of last-mile delivery can be achieved by decreasing the distance traveled for door-to-door delivery and by lowering the number of packages that are considered as missed deliveries. Innovative ideas such as in-car delivery services have been released by automotive companies, such as Volvo and GM, who have teamed up with Amazon to provide this service. This includes times where customers are not present in their cars. In such cases, the couriers will acquire a unique one-time digital key that can help unlock the customer’s vehicle for package delivery [108]. Even though several studies have been conducted on last-mile delivery and cargo-bikes [107,108,116,117], it remains challenging, especially in dense, congested areas and in harsh weather conditions.

#### 3.3.3. Smart Intersection Management Systems

According to many studies [118,119], intersections are accountable for a large number of dangerous accidents in addition to high traffic delays in urban cities. As a result, intersections have a serious impact on the efficiency of traffic management systems [120]. Therefore, CV data such as real-time heading, speed, and position are considered significant elements in assisting to optimize the traffic management systems. This will pertain to safety and operational cases, where controlling traffic signals is a necessity when different traffic streams coexist. This could be achieved with priority rules, which include stop signs, roundabouts, and right-of-way rules along with different traffic-signal approaches, such as fixed, actuated, and adaptive approaches. For instance, studies have addressed adaptive traffic control algorithms, where predictive models are integrated with the adaptive traffic control systems to have an estimate on the number of arriving vehicles and the queue length at the intersections. Even though these control strategies are able to accommodate variations in traffic demand, there exist some limitations regarding the maximization and minimization of the green-time periods. This allows for more potential efforts in enhancing the traffic operation efficiency at intersections to accommodate the variations in traffic demands effectively, and hence decreasing traffic delays [121].

Even with the existence of many studies in both signalized and non-signalized intersections [122,123,124,125], intersection traffic management remains a challenging research area in intelligent transportation systems.

#### 3.3.4. Collaboration of Smartphone Applications with Vehicles

Automotive companies including Volvo, Bosch, and many others are utilizing smartphone applications to provide new innovative services. For instance, a smart phone application can unlock the vehicle and start/stop the engine, provide access to authorized riders (e.g., in ridesharing scenarios), notify the owner of any upcoming services, and remotely check the fuel level [126,127]. Another example is the digital driver map that drivers can use to locate/reserve parking spaces [126]. The potential of these applications will provide a myriad of new innovative services within the vehicle.

Table 2 provides a summary of the applications that have been discussed in this article along with the available references to gain a better understanding of these applications.

## 4. Challenges

The following subsections identify and describe the fundamental challenges of CV technology. Recommendations and suggestions to handle these challenges are discussed in an effort to improve the future development and deployment of CVs technology.

### 4.1. Integration of Communication and Computation

The advancements in mobile cloud computing, distributed data storage, and vehicular communication technologies have played a significant role in the modeling and implementation of cooperative vehicle infrastructure systems. CVs possess onboard units that provide storage, communication, and computation functionalities, which allow these vehicles to offer cloud computing services to other connected mobile devices within their ranging scope [128]. This has given rise to innovative vehicular cloud computing from mobile cloud computing [129,130], which has received increasing attention in recent years. In this paradigm, a cooperative small-scale cloudlet is established using inter-vehicle communication between vehicles and infrastructure. Thereby, vehicles are able to offload their different computation tasks to RSUs or central cloud data centers, or they may also utilize the computing resources available within vehicles in near proximity. Other cloud computing models have been deployed to assist intelligent CVs such as cloud computing software updates and training powerful deep learning algorithms [97].

The dynamic nature of CVs poses a significant challenge regarding the practical implementation and scalability of cooperative computation. This is in addition to the unique characteristics and limitations of vehicular networks, such as fast channel fading, deterioration that may result from Doppler effects, and many others that might affect the reliability and efficiency of the transmitted data, for instance, when sending music or video files between vehicles and between vehicles and infrastructure [128]. As a consequence, computation performance in terms of reliability and efficiency is also greatly affected. This adds to the cloud-computation processing limitations, such as long processing times and high volumes of transmitted data. High latency in cloud computing relies on the wireless channel status, the network bandwidth, and the traffic congestion, thus hindering efficient real-time and reliable processing [97].

Further research is needed in designing robust and efficient vehicular cloud computation models in highly dynamic vehicular environments such as the open vehicular data analytics platform [131] and CAVBench [132] to address the QoS requirements of safety critical applications in real time. These vehicular applications require low latency and high reliability, along with secure and private data transmission. Even though there exist several research studies in vehicular cloud computing with resource management problems such as scheduling strategies, this research scope is another challenge that needs to be further explored.

### 4.2. Over-the-Air (OTA) Updates

Over-the-air (OTA) software updates are expected to be of great importance for CVs. Therefore, updates regarding the vehicle’s performance or correctness of any malfunctions that may affect the embedded software equipped within the electronic control unit (ECU) can be conducted remotely. OTA will bring many benefits to the automotive industry including OEMs as well as drivers. According to IHS [133], one of these benefits is cost efficiency, as they forecast that savings due to OTA will reach up to USD 35 billion in 2022. This has motivated and derived many automotive makers to work towards endorsing OTA updates as a key element of their vehicles. OTA can provide new software update specifications to fleet vehicles that are currently on the roads. Furthermore, OTA offers new specifications to head units and tackles any vehicle-related issues. Hence, increasing revenues and decreasing the residual value losses by allowing vendors to provide add-on missing features. To leverage the OTA benefits, OEMs and suppliers are required to design end-to-end architectures and support modular in-vehicle software that require ease-of-upgrade functionality. Additionally, the infrastructure and backend systems should be designed to handle OTA updates that could allow for add-on features specific to the system design [15].

Although remote OTA software updates are considerably beneficial to the automotive industries, since it is fast and saves money and time as the driver can have the update anytime anywhere, it still raises and pertains to significant security issues. This is because the installation process of updating to the latest software on ECU will require full accessibility to in-vehicle communication networks [133,134].

Herberth et al. [135] addressed the problem of decreasing the update time. One of the proposed solutions was to conduct parallel updates for ECUs that might reach up to 100 within an in-vehicle network. This solution has shown great impact in time reduction of the updates. However, there has been no optimal parallel scheduling algorithm that is able to utilize the full benefits of in-vehicle networks. Therefore, the authors presented a framework with high abstraction for an optimized parallel ECU software update scheduling. Hybrid and mixed-integer linear program scheduling algorithms were developed and tested against 20 ECUs in regards to both update and computing periods. Their evaluation shows that optimal parallelization can reduce the duration of reprogramming to 77% in comparison to sequential update, and their high abstraction model feature allows ease of adaptation to new vehicles’ generations. Another important aspect of OTA software update is the possibility of software failure. In such cases, vehicles should be able to retrograde to older versions in an effort of reducing potential risks, which may occur as a consequence of the software update failure [136]. In [75], the authors present SecUp, a generic model for providing secure and reliable wireless software updates. SecUp makes use of IEEE 802.11 to connect vehicles and diagnostic devices in an efficient, fast, and dependable scheme. Wireless software updates feature including parallel and partial software updates and are enabled by SecUp for high efficiency along with adapting a secure mechanism to prevent attacks and malicious threats.

Tesla makes use of wireless networks to provide remote updates from servers at the OEM location to vehicles. The use of a dedicated communication channel will not allow parallel concurrent installation on ECUs, which is considerably time consuming, as Tesla takes 20–90 min to complete the update [136]. GM unveiled a new “*digital nerve system*” that will enable OTA software updates on all vehicles. This architecture will support 4.5 terabytes of data transfer per hour [137]. Most of the OTA software update research scope was conducted to address the security breaches caused due to complete access to in-vehicle communication networks during the updating process. However, little or minimal attention has been given to the aspect of reducing the time of the OTA software updates.

### 4.3. Privacy and Security

High-data networks are usually prone to hackers and cyber–physical attacks. Without proper and valid protection in the CV environment, a vehicle’s controllers could possibly be hacked, valuable information would be stolen, and personal data would be revealed. This raises many security and privacy concerns and imposes significant threats on automated and connected vehicles with electronic actuators. Hackers can disseminate erroneous traffic information to cause congestion or can force the vehicle to suddenly stop, jeopardizing people’s lives [11,138]. Many security breach examples have proven to be life threatening, where security breaches include unauthorized data access and attacks that may result in physical disturbance in delivering the required services [139]. As a real-life example, in 2015, a Jeep Cherokee was hijacked by hacker experts Charlie Miller and Chris Valasek through exploiting Uconnect, which is the CV platform integrated within the vehicle that provides entertainment, navigation, and connectivity over the Internet [138,140]. They completely gained full control over the vehicle remotely over the cellular network and succeeded in controlling the radio station, air conditioning, and windshield wiper blades and then eventually seized control of the brakes and brought the engine to a halt. As a consequence of these security vulnerabilities, Fiat Chrysler had to recall 1.4 million Jeep Cherokee to address this issue [140].

Privacy attacks that are directed towards the vehicles’ geographical location and driving habits of the drivers are among the most threatening privacy invasions. Privacy geographical location violations of vehicles enable malicious attackers to gather time series information regarding the vehicle’s position. In this context, time series information can be procured from broadcast data sent by the vehicle or across transmitted data to service data-centers through cellular networks. Additionally, attackers can perform privacy breaches that target information regarding the driving behavior, which can help reveal private and personal data about the driver [141]. Whereas, security attacks may include information integrity threats. In this attack, cyber-attackers could be able to breach the network and succeed in eavesdropping or could change the signature tied to the data being transmitted over the wireless communication interface. Solutions to this type of threat include the employment of cryptographic-based techniques. Protected interchangeable key and public-key certificates are acquired by centralized trustworthy public key infrastructure (PKI) and are both needed for cryptographic-based methodologies. Data authentication is performed by PKIs, where certificates are dispatched to inform the receiver that the data is from a trusted transmitter. However, with the increasing numbers of CVs, authenticity and key exchange might undergo issues such as single points of failure and scalability problems [142]. Moreover, cryptographic-based approaches might not be feasible for attacks such as blackhole, wormhole, spamming, and others. However, the digital signature technique and bit commitment tactics might hinder their effect [143].

Many studies have been focused on the privacy and security of CVs with an emphasis on challenges that arise due to privacy and security breaches. For instance, in [142], the authors bring forward issues regarding attacks that target on-board sensors or network attacks on wireless communication interfaces. Those attacks might greatly affect the data correctness and integrity. Even though centralized solutions exist, they are not scalable and are unable to verify the correctness of the exchanged information. The decentralized security blockchain-based technology approach is considered promising in tackling those problems. However, the presence of permissionless linear hash chains that adapt blockchain technology approaches are not feasible, as it incorporates minimum data exchange throughput and demands immense computing costs. Therefore, the authors propose TangleCV, a decentralized methodology for protecting information exchange for CVs by employing Tangle, which is a directed acyclic graph dependent on blockchain design. In this context, their preliminary architecture of TangleCV addresses the information correctness issue along with bandwidth optimization for data integrity specifications of CVs. The authors in [144] emphasize the use of blockchain technology as one of the best technologies in terms of confidentiality and security for the control systems in real-time based environments. Inspired by the increasing growth of online taxi cab reservation services that demand highly protected, robust, and consistent information exchange, the authors presented a blockchain scheme in order to provide confidentiality and transparency among clients and taxi cab drivers. Hence, they dealt with the in-vehicle smart-sensor intrusions that can be attacked by hackers. To achieve this task, each item with respect to vehicles or Internet-of-Things (IoT) devices was traced and registered within the blockchain. Information extraction from IoT devices and storage of extracted records were conducted to ensure the client’s safety and the device’s security. Their proposed framework decreased the number of fabricated requests and the change in user’s ratings. A success rate of 79% in their proposed blockchain was noted and indicated by the simulation. Biron et al. [145] point out the importance of designing control systems that are resilient to cyber-attacks. The first phase of designing these control systems is identifying the frequency of cyber threats. The authors propose a real-time based framework that determines the denial of service of frequent threats and that hence evaluates the impact of this attack on the CV system. Their model is composed of a collection of observers developed using a sliding mode and the adaptive estimation theory. Lyapunov’s stability hypothesis was used to analyze the mathematical convergence features of the observers. Validation of the proposed methodology was conducted under a number of parametric uncertainties and various quantifying noise scenarios by simulation.

The authors in [146] propose a permission-based blockchain system to access and utilize in-vehicle data following traffic-accident scenarios for forensic analysis purposes and addressed issues that arise from storage overheads and membership-based management within the blockchain technique. Their framework integrated vehicular public key infrastructure (VPKI) to guarantee secure membership. The authors also discussed a fragmented ledger that supports both hashed and non-hashed data. The authors in [147] address acquiring traffic information from centralized-based navigation agents such as Apple Maps and Google Maps, which are mostly prone to many vulnerabilities including security and privacy attacks. These attacks can jeopardize the driver’s personal information and can track activities in real-time. Thereby, the authors present“*Traffic Chain*”, which is a decentralized two-layered blockchain-based framework that gathers traffic information by utilizing the fog and cloud computing paradigm. Their framework further integrated the long short-term memory (LSTM) deep learning algorithm, which has been evaluated through simulation to be resilient against attacks such as Byzantine and Sybil. Dorri et al. [148] design a decentralized light scalable blockchain (LSB) architecture that provides a secure overlay network between vehicles, original equipment manufacturers (OEMs), and service providers, where user privacy is protected using a changeable public key. The authors listed different applications including insurance and car-sharing services to validate their proposed architecture. Further to these efforts, a consortium named the *Mobility Open Blockchain Initiative* (MOBI https://dlt.mobi/ (accessed on 11 November 2021)) was formed by GM, BMW, Renault, Ford, IBM, Hyperledger, IOTA, and Consensys (Ethereum) to focus on promoting standards and accelerating the adoption of blockchain, distributed ledger, and related technologies in the vehicle domain.

The prominent need for a private and secure CV system will drive the research community to enhance, develop, and innovate low overhead and lightweight security and privacy algorithms against different cyber–physical attacks that are considered life threatening for vehicular environments. The aforementioned studies have addressed different security and privacy aspects to encourage automotive companies to incorporate a *“security by design”* paradigm at the early stages of vehicle manufacturing to mitigate different security and privacy attacks. Table 3 summarizes the challenges, potential research, and state-of-the-art technologies to handle these challenges.

## 5. Opportunities

In recent years, the automotive sector has witnessed rapid growth, innovations, and many technological advancements that have been targeting connected and autonomous vehicle technologies. Many opportunities lie ahead to further enhance and develop different aspects of this technology. CVs incorporate many technologies, and, hence, many opportunities can be envisioned in various aspects of CVs. For instance, innovative solutions for developing and increasing the efficiency of cloud platforms are considered a necessity to gain better insights from the huge amount of data that are produced in the CV environments. Moreover, with the high safety level that CVs provide, equal research opportunities towards innovative applications and services are widely open. One of the promising applications is the shift towards smartphone applications to assist in some of the functionalities of the vehicles. This provides more business opportunities for both automotive and telecommunication companies.

Additionally, smart commercialization is today’s new market sales tactics where stores, restaurants, and many industrial companies turn to digital commercialization instead of the traditional means in advertising their products and offers. Therefore, utilizing the data dissemination between vehicles for sending offers to drivers and using vehicles as an advertising platform will provide equal profit, opportunities, and revenues for the advertised party, since monetizing data in accordance to the driver’s preferences to provide the most convenient services and advertisements are considered promising.

Voice-recognition technologies incorporated with modern vehicles are an integral part of the in-vehicle dashboards to help drivers use their voices instead of being distracted by other means, such as texting. Even though in-vehicle voice-control-assistant technology is envisioned with various applications that assist drivers to fully interact with the vehicle to access information, such as emails or map directions by speech, many innovative voice applications are foreseen to further dominate. Hence, they will increase the safety level for drivers by keeping their eyes focused on the roadway. Machine learning algorithms and data analytics can also learn about the driver’s preferences and daily routines to offer a more personalized driving experience. Therefore, they open up new possibilities and opportunities for unique and customized applications and services.

Cooperative perception is foreseen as a potential approach that overcomes and improves the sensing-based technology limitations of CVs by allowing vehicles to exchange their local raw or processed sensor information via V2V communication to improve their visibility and field of vision. In a dense environment, vehicles may redundantly transmit the same perceived data to nearby vehicles, which results in a reduction in V2V communication reliability, inefficient use of network resources, and high network loads that lead to emergency data or critical information being lost or delayed and hence jeopardizing road safety [149,150]. Developing intelligent schemes to mitigate redundant data and improve network loads and scalability while utilizing network resources efficiently are promising research directions.

In connected environments, vehicles communicate with the surroundings to illuminate and mitigate imminent crashes. Specifically, in situations involving on-the-verge crashes with pedestrians, vehicles’ and pedestrians’ smart devices communicate with each other to alleviate this type of collision, creating the necessity for on-demand QoS and low-latency messaging in real time. The development of on-demand QoS-techniques for efficient V2P communication in risky situations has promising opportunities [151]. There are also huge opportunities for CVs to contribute to infrastructure- and environmental-sensing services, such as road roughness or estimation of the International Roughness Index, bank angle, lane-marking quality, potholes, speed bumps, snow and dust coverage, etc. [152].

Automotive radars are considered as an indispensable safety technology within today’s and the next generation of modern vehicles. The most commonly used automotive radars are designed to meet the maximum radar range regardless of the traffic density due to their operation in the high bandwidth of mm-wave frequency band and their dependence on frequency modulated continuous waves (FMCW). This may lead to a number of technical challenges including radar–radar interference in dense traffic situations when vehicles are in close proximity to one another. This is in addition to the underused time-frequency allocated resources that are directly associated with the operating range. These technical challenges will provide opportunities for future research directions [153,154].

Visible light communication technology provides new possibilities in the vehicular-based applications domain. VLC technology proposes many benefits including high data transmission (up to 10 Gbps), a low level interference range, and considerable vigor against commonly known jamming attacks in wireless communications. However, pitfalls still exist within the technology, as VLC is susceptible to noises formed by additional light sources, which makes it inconvenient in adverse weather conditions and unable to handle non-LOS situations [155,156].

The advent of the commercial roll-out of 5G cellular technology will support a wide range of ultra-low-latency tangible-access networks and connected applications. Data- and user-centric approaches were adopted in LTE and 5G cellular technologies, in an effort to meet the daunting demand of transferring huge amounts of data at high speed. New research efforts have been directed towards 6G technologies to go a step further beyond personalized communication platforms towards a fully digitized connected Internet-of-Things paradigm, where heterogeneous devices are connected including computing resources, on-road vehicles, wearable devices, and many others. In its preliminary stages, 6G will be established on terrestrial and satellite communication providing seamless global coverage [157]. Furthermore, TeraHertz-based 6G technology will offer a phenomenal user experience due to the supported ultra high quality data rates. Additionally, 6G technology will endorse a number of cutting-edge technologies including intelligent reflective surfaces (IRS), quantum computation, and many others. Extensive research should be directed to some of the technical challenges including reflection optimization in IRS-aided technology, channel estimation/adaptation in the high mobility vehicular environment, and various frequency bands’ ranges, respectively. Quantum computation will be integrated within the next generation 6G-V2X communication technology to provide high-level computation capabilities along with a security-rich V2X communication. However, technical challenges still pertain in regards to quantum computation scalability and the established quantum security frameworks’ blueprints [158].

## 6. Conclusions and Future Discussion

CVs technology brings some challenges and opens up new possibilities, applications, and services. These innovative applications and services range from the shift towards keyless vehicles to digital parking maps to smart traffic management and eco-friendly environments. This originates from the abundance of in-vehicle sensing and improvements in onboard computation processing, networking, and communication, which paves the way for innovative applications that guarantee a safer, convenient, and more environment-friendly driving experience.

This study provided a deep dive and a comprehensive review of CV technologies and shed light on key enabling technologies, such as DSRC and 5G cellular communications. A precise look over the latest status quo and industrial directions on each of these technologies was presented to the reader. We introduced and provided recommendations about the technical challenges associated with CVs technology in terms of OTA, privacy, security, integration of computation, and communication issues. We also provided analytical discussions on safety and non-safety applications and drew the attention of the research community towards open challenges and promising applications that can greatly enhance traffic efficiency and operations. Future opportunities and research directions were highlighted, which will assist CV technologies to bring a more prosperous future to humanity.

## Figures and Tables

**Table 1:** Comparison between DSRC, 5G-cellular, and Wi-Fi 6 technologies.

Features	DSRC	5G-Cellular Technology	Wi-Fi 6
Latency	Low latency and high reliability in less-congested areas [49].	Latency is considered stable in comparison to DSRC. Expected <1 ms.	2–3 ms [50].
Communication Range	300–1000 m.	Up to 2000 m.	50 m indoors and 300 m outdoors [51].
Benefits	Established and deployed technology. Have an allocated 5.9 GHz spectrum. Perform well in harsh weather conditions [52].	Maximum download data rate can reach up to 4.5 Gb/s [53]. Covers a large area and is suitable for long-range applications.	Higher spectral efficiency/channel capacity. Broader outdoor coverage.
Challenges	Short-term V2I interconnection. Low scalability in dense traffic environments. Unfairness in acquiring resources.	High communication cost. No established dedicated standard. Interference at low-level altitude. Centralized architecture of cellular networks.	Lower coverage range.

**Table 2:** Examples of Potential CVs Applications.

Application Name	Description	References
RLVW	Drivers are informed if they are about to cross a red light.	[73,74,75,78]
EEBL	Data is sent to alert drivers of hard braking performed by the vehicle in front of them.	[80,81,82,83,84]
CSW	Drivers are notified that they are about to enter a curve with high speed	[85,86,87,89]
Platooning	Vehicles/trucks maintain inter-vehicle distances and are synced (speed, routes, and braking) to achieve enhanced fuel consumption	[90,91,92,94]
Infotainment	Commercial/entertainment applications	[96]
Vehicle as a mobile service	Providing of other services beyond safety and entertainment such as localization or commercialization.	[4,97,98]
Data monetization	Monetizing data to provide new innovative services.	[99,100,101,102,103,104,105]
Last-mile delivery	Tackles efficient delivery of packages/parcels	[107,108,109,110,112,113,114,115,116,117]
Smart intersection management	Intersection management at both signalized and non-signalized intersections to increase efficiency and decrease traffic delays.	[118,119,120,121,122,123,124,125]
Collaboration of smartphone applications with the vehicle	Integrating smartphone applications to aid in some of vehicle’s functionality such as replacing keys with smartphone application.	[126,127]

**Table 3:** Summary of the challenges in CV technology.

Challenges	Potential Research	State-of-the-Art Technology	References
Integration of communication and computation	Vehicular cloud computing with resource management problems	Vehicular mobile edge computing	[97,128,129,130,131,132]
Over the Air (OTA)	Methodologies and techniques to decrease the OTA update duration time	Server/client	[133,134,135,136]
Privacy and security	Need for lightweight and low overhead privacy and security algorithms	Blockchain technology	[139,140,141,142,143,144,145]

## Data Availability

Not applicable.
